# Growth Hormone and the Human Hair Follicle

**DOI:** 10.3390/ijms222413205

**Published:** 2021-12-08

**Authors:** Elijah J. Horesh, Jérémy Chéret, Ralf Paus

**Affiliations:** 1 Dr. Philip Frost Department for Dermatology and Cutaneous Surgery, University of Miami, Miami, FL 33136, USA; elijjonathan@med.miami.edu (E.J.H.); jpc219@med.miami.edu (J.C.); 2 Monasterium Laboratory, D-48149 Münster, Germany; 3 Centre for Dermatology Research, NIHR Manchester Biomedical Research Centre, University of Manchester, Manchester M13 9PT, UK

**Keywords:** growth hormone, insulin-like growth factor-1, somatotropic axis, hair follicle

## Abstract

Ever since the discoveries that human hair follicles (HFs) display the functional peripheral equivalent of the hypothalamic-pituitary-adrenal axis, exhibit elements of the hypothalamic-pituitary-thyroid axis, and even generate melatonin and prolactin, human hair research has proven to be a treasure chest for the exploration of neurohormone functions. However, growth hormone (GH), one of the dominant neurohormones of human neuroendocrine physiology, remains to be fully explored in this context. This is interesting since it has long been appreciated clinically that excessive GH serum levels induce distinct human skin pathology. Acromegaly, or GH excess, is associated with hypertrichosis, excessive androgen-independent growth of body hair, and hirsutism in females, while dysfunctional GH receptor-mediated signaling (Laron syndrome) is associated with alopecia and prominent HF defects. The outer root sheath keratinocytes have recently been shown to express functional GH receptors. Furthermore, and contrary to its name, recombinant human GH is known to inhibit female human scalp HFs’ growth ex vivo, likely via stimulating the expression of the catagen-inducing growth factor, TGF-β2. These limited available data encourage one to systematically explore the largely uncharted role of GH in human HF biology to uncover nonclassical functions of this core neurohormone in human skin physiology.

## 1. Introduction

The human hair follicle (HF) behaves as a neuroendocrine organ, even when isolated from systemic (blood flow, peripheral nervous system) stimuli, and shows hormone and receptor expression analogous to several central pituitary neuroendocrine axes [1,2]. Namely, the synthesis, secretion, and regulation of hormones of the hypothalamus-pituitary-adrenal (HPA) axis have been documented in human scalp HFs ex vivo. In the absence of systemic connections, cultured human scalp HFs express and respond to corticotropin-releasing hormone (CRH) and adrenocorticotropic hormone (ACTH), resulting in the HF synthesis of cortisol and activation of classical neuroendocrine feedback loops. Just as in the central HPA axis, the expression of pro-opiomelanocortin (POMC), the precursor for ACTH, α-MSH, and β-endorphin, is upregulated by CRH, while cortisol downregulates intrafollicular CRH protein synthesis [3]. All three POMC-derived peptides listed above regulate HF melanogenesis [4], while insufficient HF synthesis of melanotropic HPA axis hormones may contribute to HF greying [5].

HFs are also extra-pituitary sources of prolactin [6] and thyrotropin-releasing hormone (TRH) [7]. TRH and estradiol both regulate prolactin and prolactin receptor expression in human HFs in a similar manner as they do in the pituitary gland [8]. However, the HF expression of prolactin also underlies distinct controls, namely, it is not regulated by dopamine (as in the pituitary gland) but by substance P and the proinflammatory cytokine interferon-gamma [9]. Human HFs also express functional thyrotropin receptors [10], whose stimulation promotes intrafollicular mitochondrial activity and biogenesis [11].

Yet, the role of another key neurohormone, growth hormone (GH, somatotropin), in human HF biology remains insufficiently explored. After providing basic background on general GH biology, we delineate in the current review clinical and experimental evidence in support of GH as a potentially important regulator of human HF physiology. We argue that the limited available data encourages one to systematically dissect the role of GH in human HF biology in order to uncover nonclassical functions of this core neurohormone in human skin physiology and to develop novel GH or GH receptor-targeting neuroendocrine strategies for the therapeutic manipulation of hair loss (effluvium, alopecia) and unwanted hair growth (hirsutism, hypertrichosis).

## 2. The Hypothalamus-Pituitary-Somatotropic Axis

**Figure 1:**
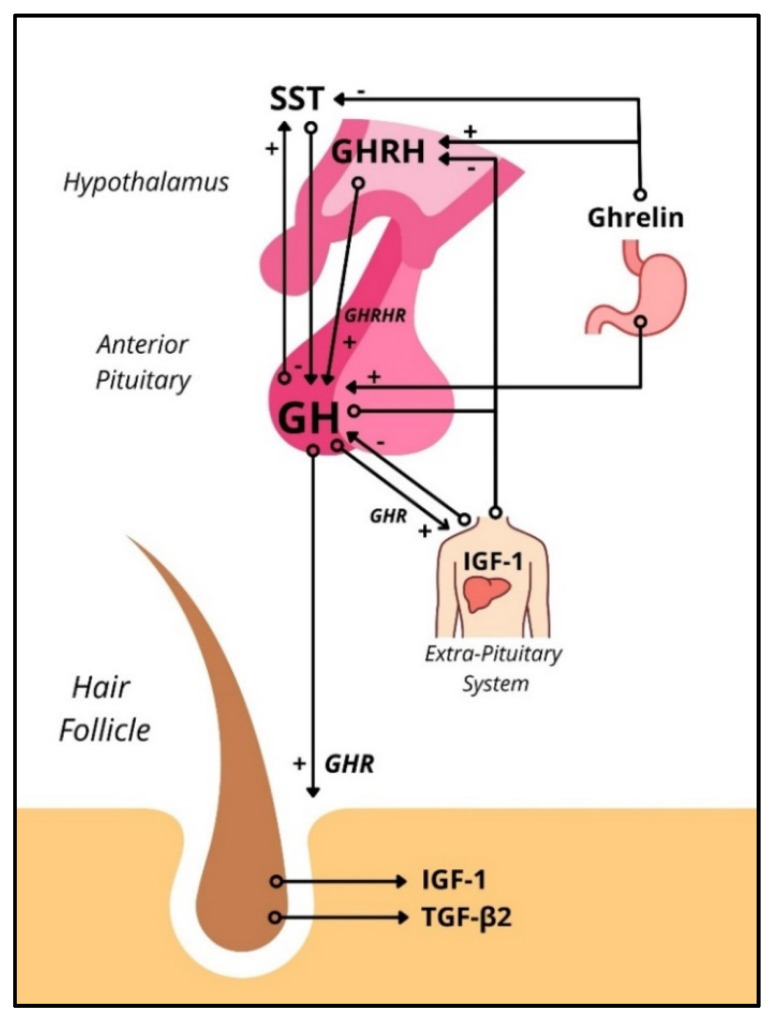
Schematic representation of the HPS axis interacting with the human hair follicle. In the HPS axis, GHRH acts on the GHRHRs in somatotropic cells of the anterior pituitary to release GH systemically, which interacts with GHRs systemically, including in the HF. GHR activation increases IGF-1 transcription, which exhibits negative feedback on GH synthesis in the anterior pituitary and GHRH synthesis in the hypothalamus. GHRs have been found in the human HF. Stimulation of GHRs in HFs ex vivo has shown to inhibit hair growth in female human scalp HFs via upregulation of TGF-β2 (Alam, et al., 2019). HPS = hypothalamic-pituitary-somatotropic, GH = Growth Hormone, GHR = Growth Hormone Receptor, GHRH = Growth Hormone-Releasing Hormone, GHRHR = Growth Hormone-Releasing Hormone Receptor, IGF-1 = Insulin-like Growth Factor-1, TGF-β = transforming growth factor β.

The hypothalamus-pituitary-somatotropic (HPS) axis refers to the neuroendocrine control of GH secretion and its downstream signaling. Growth hormone-releasing hormone (GHRH) produced in the hypothalamus upregulates GH gene expression and stimulates the release of GH from pituitary somatotrophs. Somatostatin (SST), also produced in the hypothalamus, inhibits GH release (but not GH synthesis) in pituitary somatotrophs. Both hormones act on the pituitary via the adenohypophyseal portal venous system. The orexigenic gastric peptide ghrelin stimulates hypothalamic GHRH secretion and pituitary GH release [12]. GH acts on peripheral cells in virtually every human tissue directly through the growth hormone receptor (GHR) (Figure S1) and indirectly through the insulin-like growth factor 1 (IGF-1). The downstream signaling from activating the GHR varies by cell population but commonly involves the JAK2-STAT1/3/5 and/or MAPK pathways. Downstream, suppressors of cytokine signaling (SOCS) are known to inhibit GHR signaling effects. Interestingly, SOCS are upregulated by estrogen, which may cause sex-dependent differences when studying the HPS [13]. IGF-1, usually upregulated by peripheral GH signaling, inhibits GH secretion via negative feedback at the pituitary and hypothalamic levels [12] (Figure 1).

The somatotrophic axis is closely linked to sleep and the circadian rhythm. GHRH has sleep-promoting effects, and GH secretion occurs in a pulsatile fashion, with maximal levels occurring after the onset of slow-wave sleep. Abnormal circadian rhythm disorders like narcolepsy are associated with abnormalities in the HPS axis [14]. Interestingly, the HF demonstrates circadian-dependent clock gene activity in the absence of central clock influences ex vivo [15]. PER-1 and BMAL-1 were both shown to regulate human HF cycling, as well as melanogenesis [16]. The peripheral clock activity has also been shown to be modulated by neurohormones like thyroxine [17], suggesting that other neurohormones like GH may also modulate HF biology directly or indirectly via modulation of clock genes. GH serum levels correlate with serum estradiol levels, with higher concentrations found in young people when compared with older people, as well as in females when compared to males. The bulk of GH secretion in males occurs during the night, whereas in females, nighttime secretion of GH corresponds to a smaller fraction of total daily GH secretion [18], which confirms that GH and clock genes are in direct connection.

### 2.1. Growth Hormone-Releasing Hormone

GHRH belongs to the secretin family of peptide hormones, which includes glucagon, secretin, vasoactive intestinal polypeptide, and others [19]. GHRH undergoes rapid enzymatic degradation in the blood via dipeptidyl peptidase IV [20] and therefore has negligent serum levels. While the primary function of GHRH is considered to be regulating pituitary GH synthesis and release, GHRH has been observed to be produced and have autocrine/paracrine effects in extra-pituitary tissues (Table 1) that stimulate cell proliferation and inhibit apoptosis [19]. GHRH has been shown to promote wound healing by stimulating proliferation and survival of human dermal fibroblasts via signaling of the GHRH receptor [21].

The GHRH receptor (GHRHR) is a class II B GPCR found on the cell membrane of the pituitary somatotroph. Activating this receptor stimulates the exocytosis of GH and transcription of the *GHRHR* gene [20]. GHRHR activation is also vital to somatotroph cell proliferation via βγ subunit-mediated activation of Ras-MAP kinase and ERK phosphorylation. Extrapituitary GHRH activity is mediated by GHRHR and its splice variant type 1 (SV1), found in several cancerous and noncancerous human tissues, including apocrine glands and dermal fibroblasts (Table 1) [21,25,26]. GHRH signaling, via the GHRHR and the SV1, has been implicated in the growth of human apocrine tumors and metastatic melanoma [21,27]. GHRHR antagonists have been shown to inhibit cancer growth in vitro and in vivo and have anti-inflammatory and antioxidative effects [28,29].

### 2.2. Insulin-like Growth Factor-1

IGF-1, also called somatomedin-c, is a 70 amino acid protein with structural homology to pro-insulin. Gene expression of *IGF-1* has traditionally been thought to be regulated by GH stimulation primarily in the liver. However, IGF-1 is expressed in most, if not all, tissues. GH stimulation is known to regulate both IGF-1 and many IGF-binding proteins (IGFBPs) [30].

IGF-1 receptor (IGF1R) is a transmembrane tyrosine kinase receptor consisting of two α subunits and two β subunits synthesized from a single mRNA precursor. Activating IGF1R leads to autophosphorylation of tyrosine kinases, leading to activation of several downstream signaling pathways, all of which stimulate the growth and proliferation of different cell populations [30]. For example, IGFs stimulate fibroblast proliferation, survival, migration, and production of growth factors like platelet-derived growth factors A and B [31]. IGF-1 is also known to be the most potent anagen prolonging growth factor in HFs [31,32]. In addition, IGF1Rs were found to be expressed in the hair matrix and outer root sheath keratinocytes of human scalp HF, where their signaling promotes proliferation and maintains the anagen phase [23,24,32,33]. 

Human fibroblast culture in vitro and human skin ex vivo has been shown to increase the expression of IGF-1/-2 and their receptors in response to GH and other factors [23,24]. IGF-1 plays a critical role in both skin and hair physiology, so it is not surprising to observe GH influencing hair growth.

## 3. Ex Vivo, rGH Induces Premature Catagen Entry in Female Hair Follicles 

The known clinical hair phenotype associated with Laron syndrome or reduced GH serum levels (Table 2), which is also associated with decreased expression of IGF-1, would have led one to expect that the growth of organ-cultured human scalp HFs would be promoted by GH treatment. Unexpectedly, GH-treated microdissected human female scalp HFs showed premature catagen induction, most probably mediated via the upregulation of the potent catagen-inducting growth factor, TGF-β2 [23]. Even though IGF-1 expression in the outer root sheath keratinocyte was also upregulated, as expected, the overall increase of TGF-β2 expression in response to GH treatment may have been dominant over IGF-1, resulting in the observed growth inhibition in female HFs.

However, these phenomena may not necessarily reflect only the direct effects of GHR stimulation within the HF itself. They might represent the overall HF response to complex neuroendocrine changes associated with excessive or insufficient GH/GHR-mediated signaling. For example, chronic excessive GH signaling interferes with insulin and creates GH-induced insulin resistance [34]. Accordingly, many cutaneous findings (listed in Table 2) are found both in settings of insulin resistance and GH excess.

**Table 2:** Well-documented cutaneous manifestations of GH excess and deficiency in human skin. First listed is growth hormone (GH) excess, leading to acromegaly or gigantism, as seen in somatotroph adenoma of the anterior pituitary, neurofibromatosis-1, McCune Albright syndrome, multiple endocrine neoplasia type 1, Carney complex, and others. Then listed is growth hormone deficiency, as seen in Noonan syndrome, Turner syndrome, Prader–Willi Syndrome, and Laron syndrome, referenced from Kanaka-Gantenbein et al., 2016.

Condition	Cutaneous Manifestation	Reference
GH excess	Hypertrichosis	[35]
	Hirsutism	
	Cutis verticis gyrata	
	Acrochordons	
	Lentiginous spots	
	Melanocytic nevi	
	Acanthosis nigricans	
	Acne	
	Seborrhea	
	Hyperidrosis	
GH deficiency	Alopecia	[35,36]
	Frontal hairline recession	
	Telogen effluvium	
	Dryness	
	Thinner dermis	
	Hypopigmentation	
	Hypohidrosis	
SST Therapy	Reversible scalp hair loss	[37,38,39,40]
Low IGF-1 levels	Hair loss	[41]
GHRH deficiency	No hair loss; delayed pigmentation	[42]

## 4. Cutaneous Effects of Excessive or Reduced GH Receptor-Mediated Signaling Levels

Numerous extrapituitary tissues and cells express mRNA and protein for GH, GHRH, along with their receptors, including the skin [22,43] and HF [23], but it remains unknown whether human skin and its appendages transcribe and translate the *GH* and *GHRH* genes in vivo (Table 1). Most of what we currently know about the effects of GHR-mediated signaling arises from clinical observations in patients with excessive or insufficient serum levels of GH or defective GHR-mediated signaling.

**Figure 2:**
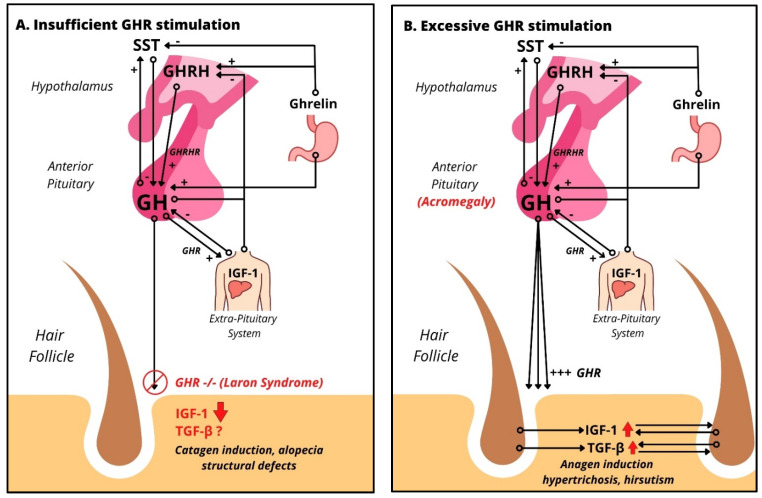
Impact of excessive and insufficient GHR stimulation on human scalp HFs: (**A**) HPS axis in the case of absent GH signaling, like Laron’s syndrome, leading to almost absent levels of IGF-1 due to absent GHR stimulation. Clinical findings include alopecia, frontal hairline recession, and structural defects. (**B**) HPS axis in the case of GH excess, like acromegaly, which upregulates both IGF-1, an anagen promoter, and TGF-β, a catagen promoter. Clinically, acromegaly patients show increased hair growth and hirsutism.

Pathologies leading to GH deficiency, like Noonan Syndrome, Turner Syndrome, and Prader–Willi syndrome, are associated with alopecia, telogen effluvium, and frontal hairline recession [35]. These syndromes are also associated with hypogonadism, which is also known to be associated with alopecia [44]. However, the interplay of androgens and GH on hair pathology is unknown and needs to be kept in mind. Male patients with GH deficiency of any cause were found to have reduced sweating [35]. These clinical findings clearly suggest that the HF and pilosebaceous unit [45,46] is a target for GH and can serve as a model system for studying how GH impacts a human mini-organ model as previously shown for other hormones (i.e., TRH, TSH, prolactin, CRH, ACTH). In this context, Laron syndrome, characterized by a loss of function mutation in the growth hormone receptor gene, leading to high levels of GH combined with low levels of IGF-1 [47], is particularly instructive. Laron syndrome is associated with sparse hair growth, various degrees of alopecia, and frontal hairline recession (Figure 2A). Structural defects are found under microscopy such as grooving, tapered hair, pili torti and canaliculi, and trichorrhexis nodosa [48] as well as hypotrichosis [49] (Table 2). Recently, Laron syndrome has been mimicked in porcine models with *GHR* knockout mutations [50]. Both humans and the porcine model develop juvenile hypoglycemia with preservation of glucose tolerance and the development of normoglycemia with the onset of puberty [51]. Simulating GHR deficiency in organ-cultured human HFs by knocking down *GHR* using our established gene silencing methodology by transient siRNA transfection ex vivo [32,52,53] should instructively complement the use of this porcine model. 

On the contrary, conditions of GH excess or deficiency have well-documented cutaneous manifestations (Table 2). Increased plasma GH level in burn patients leads to improved re-epithelialization, increased granulation tissue, and reduced healing time [54]. Conditions with excess GH result in acromegaly in adulthood, and gigantism in childhood (before the epiphyseal growth plates fuse). Clinically, the leading cause of GH excess is a GH-producing pituitary adenoma (incidence/prevalence: 0.4–1.1 cases per 100,000/4–13 cases per 100,000 [55]), which occurs much more rarely than prolactin-secreting pituitary adenomas. McCune Albright syndrome, neurorofibromatosis-1, multiple endocrine neoplasia type 1, and Carney complex, which can also be associated with excessive GH serum levels, are even more rarely encountered orphan diseases [35]. Despite their rarity, the skin abnormalities seen in these diseases (Table 2) provide important clinical pointers to the overall net impact of excessive GH serum levels on human skin and skin appendages in vivo. Excess GH is associated with hypertrichosis and hirsutism (Figure 2B), as well as hyperhidrosis and increased sebum production [35]. 

Supplementing recombinant human growth hormone (rGH) may be key to therapies for encouraging wound healing and preventing or reversing aging-related damage. Treating elderly men with rGH has led to an increase in skin thickness [56]. Increased plasma GH in burn patients leads to improved epithelialization, increased granulation tissue, and reduced healing time [54]. Recombinant GH in human skin mice models has been shown to accelerate healing in pressure ulcer wounds [57]. Moreover, a large meta-analysis study suggested that rGH treatment may be used in the treatment of diabetic foot ulcers in humans [58]. Indeed, GH is known to promote cell proliferation, stimulate immune cells, and promote angiogenesis [57,59], all of which are known to be deficient in DFU patients. 

## 5. Other Skin Phenotype Changes Associated with Signaling Abnormalities in the Hypothalamic-Pituitary Somatotropic (HPS) Axis

SST analogues are used in therapies for several pathologies, including Merkel cell carcinoma, pancreatic endocrine neoplasms, and pituitary adenomas [60,61]. The use of SST analogues in therapies has been associated with scalp hair loss that resolves with discontinuation of treatment [37,38,39,40].

Classically, SST downregulates GHRH and GH signaling, which decreases IGF-1 signaling downstream. Decreased IGF-1 levels in dermal papillary fibroblasts of the hair follicle are found in balding scalp follicles when compared to nonbalding scalp HFs [62]. Furthermore, low circulating IGF-1 levels were associated with hair loss in middle-aged women [41]. In one study observing patients post transsphenoidal adenomectomy, 54% of patients who had acromegaly experienced hair loss 3 to 6 months postoperatively, compared to 6% of patients who had nonfunctional adenomas [36]. This study also showed hair loss was more common in patients cured by surgery than in non-cured patients, i.e., hair loss was more common in patients that experienced acute decreases in GH and/or IGF-1, behaving as a relative insufficiency [36]. More interestingly, topical liposomal IGF-1 was associated with more rapid hair growth and thicker hair in a hamster model [63]. 

In mice, GHRH treatment was found to reverse age-related changes, increasing the thickness of the epidermis and dermis, increasing moisture content, and improving the morphology of the skin tissue and collagen fibers [64]. GHRH deficiency in a Brazilian cohort showed delayed pigmentation, and reported to have youthful hair and no alopecia, even with profoundly decreased serum GH and IGF-1 levels [42]. This cohort aligns with GH acting as an inhibitor of hair growth previously seen ex vivo in female donors [23]. This population did show wrinkly skin, which implicates the HPS axis’ role in age-related damaging of human skin even more.

## 6. Major Open Questions

The surprising hair growth-inhibitory results reported above ex vivo, including the upregulation of TGF-β2, question conventional concepts that the direct stimulation of GHR in human peripheral tissues always has a growth-stimulatory effect. It also raises the question to which extent GH/GHR-mediated signaling has tissue- and context (sex)-dependent outcomes in human skin and its appendages. We know that a strong positive correlation exists between excess GH levels and insulin resistance and that both higher GH serum levels and insulin resistance are found in females. This may, at least partially, explain why the upregulation of IGF-1 after GH treatment in female scalp HFs ex vivo did not prolong hair growth. One possibility to answer this question might be to investigate if the same GH concentration tested in female HFs prolongs the anagen phase in male scalp HFs via prominently increasing IGF-1 over any potential increase of TGF-β2 expression. Clinically, primary decreases in GH and IGF-1 have been associated with hair loss and alopecia [36], while decreases in GH and IGF-1 due to GHRH deficiency have not [42]. Furthermore, hair growth stimulation has been seen in acromegaly patients [35]. Understanding how hair and skin respond to GH and GHRH stimulation separately may be key in understanding these discrepancies. Dissecting the effects of GH stimulation in human HF and skin organ culture [65,66,67,68] in the presence/absence of GHR siRNA, followed by laser capture microdissection-based RNAseq analysis of defined HF and skin compartments or single-cell RNAseq, should help to clarify which cell population(s) in human skin are most receptive to GHR stimulation. These studies can also shed light on how they differ in their target genes and signaling pathways. Furthermore, GH signaling in the skin and HF still needs to be further characterized in the context of GHRH, SST, and IGF-1 expression, with any of their potential negative feedback mechanisms. Clinically, GH excess and deficiencies are often accompanied by other hormonal abnormalities, such as hypothyroidism and hyperprolactinemia, both of which are also known to affect hair growth [6,7,8,10,69,70,71]. It will then be important to understand how these GH effects are amplified or hampered by other hormone profiles (e.g., prolactin, TRH, TSH). 

Understanding the growth hormone’s effect on the HF and skin and its related signaling pathways is key to understanding future clinical therapies for dermatopathology. It might be interesting to investigate the expression levels of the different members of the HPS axis, keeping in mind potential sex differences in healthy skin and HFs as well as in some hair growth disorders (telogen effluvium, female/male pattern hair loss, alopecia areata). Moreover, since GH influences the expression level of IGF-1 and TGF-β2, it would be interesting to evaluate the impact of GH/GHR signaling on the hair follicle immune privilege (and consequently in alopecia areata) as both growth factors are well-known immune privilege guardians. This raises the question regarding the potential insensibility of the hair matrix and outer root sheath keratinocytes to some immune privilege guardians under excessive GH stimulation, and if that contributes to a patient’s susceptibility to hair disorders, such as alopecia areata, with excessive GH stimulation. 

The effect of GHRH and GH on hair physiology and wound healing should be further explored as well. GH and GHRH have both shown wound healing properties ex vivo and shown to have a strong effect on human dermal fibroblast proliferation and differentiation [21]. Robust clinical trials with rGH or rGHRH have yet to be done regarding wound healing in humans.

This physiological loss of the HPS axis hormones with age may be a key player in the aging process. Restoring GHRH and GH signaling could very well be a key player in anti-aging therapies to inhibit or reverse age-related damage of the skin. The hormones of the HPS axis are known to decrease with age, and mouse models have found anti-aging properties with treatment of GHRH via reduction of malondialdehyde and matrix metalloproteinases [64]. 

Recombinant growth hormone is very well-established and is safely used therapeutically [72]. GH and GHRH have already been shown to have physiological effects on catagen promotion, carcinogenesis, and wound healing. Understanding the potential effects of the somatotrophic hormones in the HF can further help regulate hair cycling and hair pathologies and guide novel therapies in wound healing and cancer. Indeed, as suggested by our study [23], a slight change in GH levels may have a dramatic effect on hair growth, suggesting GH levels and GHR stimulation needs to be fine-tuned and tightly regulated. It might then be essential to measure with precision GH levels (not only serum levels) to avoid unwanted effects, suggesting that GH disorders might require personalized treatment.

## 7. Conclusions

The human HF has been shown to express a wide array of neurohormones and even display negative feedback mechanisms that mirror central neurohormone axes.

Both GH release and the HF exhibit circadian-dependent regulation that may be interdependent. 

Pathological GH serum levels produce profound clinical effects on hair. Excess GH levels, and therefore excess GHR stimulation and excess IGF-1 levels are associated with hypertrichosis and hirsutism. Absent GHR stimulation, and thus severely decreased IGF-1 levels, is associated with alopecia, telogen effluvium, frontal hairline recession, as well as severe HF structural changes like pili torti et canaliculi and trichorrhexis nodosa.

GHRs are found in virtually every human tissue and prominently in the HF. Stimulation of the GHR may have a profound effect on hair growth. Ex vivo, female human scalp HFs were inhibited by GH stimulation, suggesting a complex sex-dependent interaction between hair growth and GH stimulation.

Further investigation of how GH and GHR stimulation affect hair follicle biology can guide feasible treatment options for different hair disorders.

## Figures and Tables

**Table 1:** Extrapituitary GH, GHR, GHRH, and GHRHR localization in humans. Extrapituitary findings of *GH* mRNA and protein, *GHR* mRNA and protein, *GHRH* mRNA and protein, and *GHRHR* and its splice variant 1 (SV1) mRNA and protein.

Molecule	Organs	Cell Type, Condition	Reference
*GH* mRNA	Skin	Primary Human dermal fibroblasts, in vitro	[22]
	Immune system	Human, in vivo	[22]
	Testis	Human, in vivo	[22]
	Ovary	Human, in vivo	[22]
	Uterus	Human, in vivo	[22]
	Mammary gland	Human, in vivo	[22]
GH protein	Bone	Human, in vivo	[22]
	Muscle	Human, in vivo	[22]
	Lymphoid tissue	Human, in vivo	[22]
	Brain	Human, in vivo	[22]
	Eye	Human, in vivo	[22]
	Testis	Human, in vivo	[22]
	Ovary	Human, in vivo	[22]
	Salivary gland	Human, in vivo	[22]
	Pancreas	Human, in vivo	[22]
	Liver	Human, in vivo	[22]
	Kidney	Human, in vivo	[22]
	Colon	Human, in vivo	[22]
	Stomach	Human, in vivo	[22]
	Lung	Human, in vivo	[22]
	Heart	Human, in vivo	[22]
*GHR* mRNA ^1^	Human hair follicles	Human, in vivo	[23]
GHR protein ^1^	Healthy female scalp skin	Human, in vivo	[23]
	HF epithelium	Human, in vivo	[23,24]
	ORS keratinocytes	Human, in vivo	[23,24]
	Dermal fibroblasts	Human, in vivo	[23,24]
	Sebocytes	Human, in vivo	[23,24]
	Melanocytes	Human, in vivo	[23,24]
	Matrix keratinocytes	Human, in vivo	[23,24]
*GHRH* mRNA	Placenta	Human, in vivo	[19]
	Ovary	Human, in vivo	[19]
	Testis	Human, in vivo	[19]
	Malignant cells	Human, in vivo	[19]
GHRH protein	Myocardium	Human, in vivo	[19]
	Lymphocytes	Human, in vivo	[19]
	Testis	Human, in vivo	[19]
	Ovary	Human, in vivo	[19]
	Endometrium	Human, in vivo	[19]
*GHRHR/SV1* mRNA	Non-Hodgkin’s lymphoma	Human, in vivo	[19,25]
	Glioblastoma	Human, in vivo	[19,25]
	Kidney	Human, in vivo	[19,25]
	Liver	Human, in vivo	[19,25]
	Lung	Human, in vivo	[19,25]
	Prostate	Human, in vivo	[19,25]
GHRHR/SV1 protein	Prostate	Human, in vivo	[19,25]
	Apocrine Glands	Human, in vitro	[26]
	Dermal fibroblasts	Human, in vitro	[21]

^1^*GHR* mRNA and protein are found in almost every human tissue, so only relevant hair follicle and skin cell populations are listed.

## Supplementary Materials

The following are available online at https://www.mdpi.com/article/10.3390/ijms222413205/s1.
