# Supplementary figures and images for: Growth Hormone and the Human Hair Follicle
Source: Int J Mol Sci. 2021 Dec 8;22(24):13205. doi: 10.3390/ijms222413205 (PMC8706217; doi:10.3390/ijms222413205)

**Chromosome**

**17q22**

**GH1**

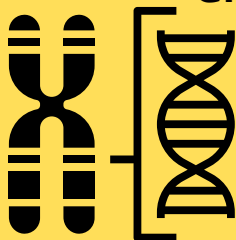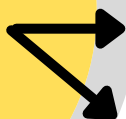

**20 kDa GH**

**22 kDa GH**

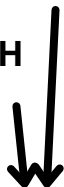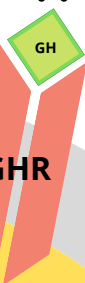

**SOCS**

**JAK2**

**STAT1**

**STAT3**

**STAT5**

**SFK**

**RAS**

**MEK**

**ERK1/2**

**MAPK**

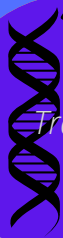

*Transcription*

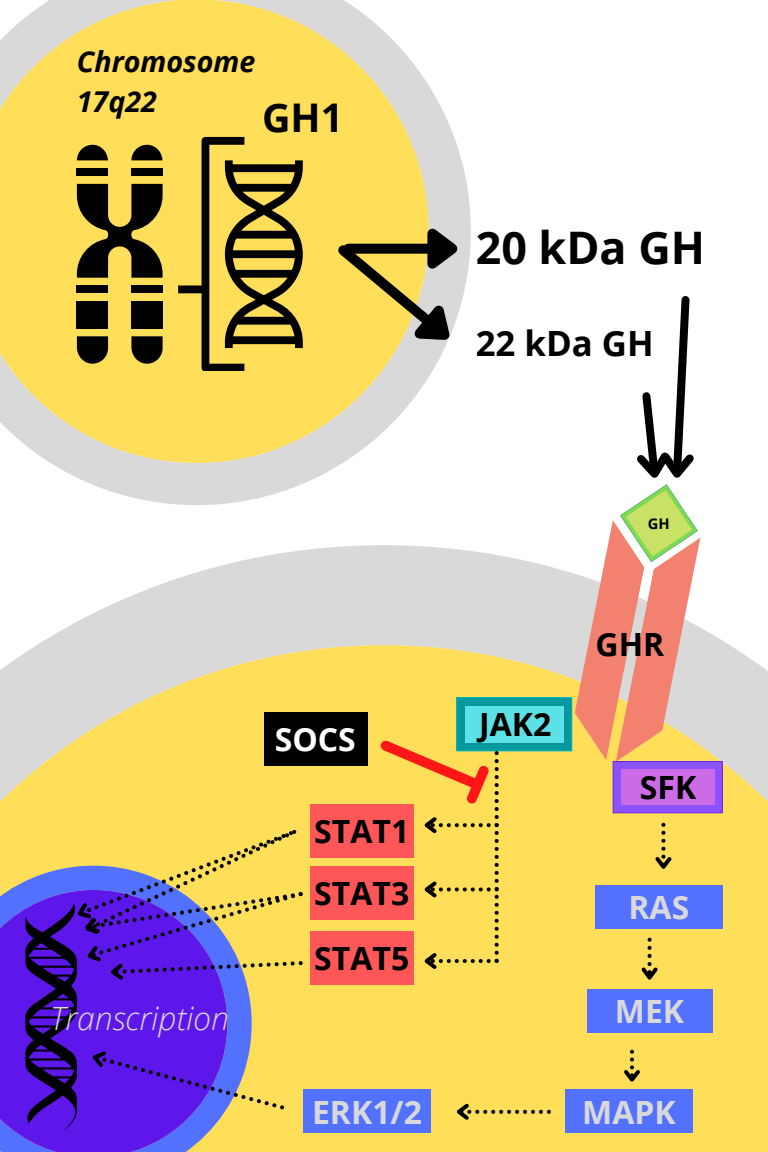

Supplement: Supplementary file 1 [file ijms-22-13205-s001.zip › ijms-1375009-supplementary.pdf]
